# Salinity-driven adaptations and evolution of DNA viruses in estuarine-coastal ecosystems

**DOI:** 10.1128/msystems.00354-26

**Published:** 2026-05-18

**Authors:** Wenqing Shi, Lu Liu, Lilin Wu, Xiaomeng Wang, Yongyi Peng, Xinyue Liu, Chengpeng Li, Jinxin Xu, Ziqi Wu, Xiyang Dong, Qiang Zheng

**Affiliations:** 1 State Key Laboratory of Marine Environmental Science, Institute of Marine Microbes and Ecospheres, College of Ocean and Earth Sciences, Xiamen University 12466, Xiamen, China; 2 Fujian Key Laboratory of Marine Carbon Sequestration, Xiamen University 12466, Xiamen, China; 3 RD3 Marine Ecology, RU Marine Symbioses, GEOMAR Helmholtz Centre for Ocean Research Kiel, Kiel, Germany; 4 Department of Ocean Science and Engineering, Southern University of Science and Technology 255310, Shenzhen, China; 5 Key Laboratory of Marine Genetic Resources, Third Institute of Oceanography, Ministry of Natural Resources, Xiamen, China; 6 Department of Microbiology, Biomedicine Discovery Institute, Monash University, Clayton, Victoria, Australia; 7 Key Laboratory of Advanced Marine Materials, Key Laboratory of Marine Environmental Corrosion and Bio-fouling, Institute of Oceanology, Chinese Academy of Sciences, Qingdao, China; Ocean University of China, Qingdao, Shandong Province, China

**Keywords:** estuarine-coastal environments, virus, salinity adaptation, auxiliary metabolic genes, microdiversity

## Abstract

Salinity gradients drive microbial diversity and evolution in estuarine-coastal ecosystems, yet viral adaptation remains less well understood. We used metagenomics to study viral adaptation and functions in three representative estuarine-coastal regions in China. Our results reveal salinity-associated adaptations in DNA viruses, with viruses enriched in medium- to high-salinity environments exhibiting higher frequencies of acidic isoelectric points and charged amino acids compared to those enriched in low-salinity environments. Viral genomes encode diverse genes related to ion transporters and organic osmolyte metabolism, suggesting potential roles in osmotic stress responses. Viral microdiversity also varied systematically along the salinity gradient, indicating reduced genetic variation and stronger purifying selection under more saline conditions. Furthermore, we identified diverse AMGs linked to nutrient cycles, with salinity-driven enrichment revealing viral roles in host metabolism. Overall, our findings highlight salinity as a key driver of viral evolution and functional potential in estuarine-coastal ecosystems, providing new insights into how viruses adapt to environmental gradients.

**IMPORTANCE:**

Salinity is a defining environmental gradient in estuarine-coastal systems, yet its role in shaping viral molecular evolution remains poorly understood. By integrating metagenomes, viromes, and metatranscriptomes across three estuaries, this study demonstrates that salinity exerts a strong and consistent imprint on DNA viruses. Increasing salinity selects for viral genomes encoding ion-transport and osmolyte-related proteins and drives systematic shifts in viral proteome composition toward osmoadaptive physicochemical properties. At the population level, higher salinity is associated with reduced viral microdiversity and stronger purifying selection, indicating constrained evolutionary space under osmotic stress. Viral auxiliary metabolic gene repertoires are structured along salinity gradients, with functional differentiation in carbon, nutrient, and nucleotide metabolism. Together, these findings identify salinity as a key evolutionary filter linking viral physiological adaptation, evolutionary dynamics, and functional potential in estuarine and coastal ecosystems.

## INTRODUCTION

Estuarine-coastal environments, situated at the interface between terrestrial and marine ecosystems, play critical roles in facilitating the exchange of materials and energy (1, 2). These regions are among the most biologically productive on Earth, characterized by unique hydrological conditions and strong salinity gradients influenced by river discharge, tidal fluctuations, and various climatic factors (2, 3). Salinity is a key abiotic factor shaping microbial community composition and diversity in these regions (4). The transition from freshwater to marine conditions introduces substantial osmotic stress, which has shaped microbial traits and led to the evolution of distinct osmoadaptation strategies. Microorganisms adapt to salinity mainly through two osmoadaptation strategies: the salt-in strategy, involving intracellular accumulation of inorganic ions (e.g., KCl), and the salt-out strategy, which maintains low intracellular salt concentrations by synthesizing or importing compatible solutes (5, 6).

Viruses are the most abundant biological entities in marine ecosystems and play vital roles in shaping microbial community dynamics and driving biogeochemical processes through host lysis and metabolic reprogramming (7, 8). Given the strong influence of salinity on microbial communities and adaptation strategies, it is also expected to shape viral communities and function. Previous studies have shown that salinity strongly shapes viral communities in estuarine and marine ecosystems (9, 10). Viral assemblages often shift markedly along freshwater-marine gradients, reflecting changes in microbial host communities (9, 10). However, it is unclear whether viruses display similar ecological boundaries between freshwater and marine environments as observed for their microbial hosts (11, 12).

As mobile genetic elements, viruses carry diverse functional genes, including auxiliary metabolic genes (AMGs) in the broad sense, such as those related to nutrient cycling, which collectively influence host metabolism, environmental adaptation, and gene flow within microbial communities (13, 14). Previous studies have shown that estuarine viruses possess diverse AMGs (10, 15, 16). As key regulators of carbon metabolism along salinity gradients, they show functional potential via carbohydrate-active enzymes (CAZymes), contributing to organic matter degradation across the salinity gradient (17). Estuarine viral communities are also enriched in AMGs related to nutrient metabolism, transport, and host growth regulation, potentially enhancing host metabolism and environmental tolerance (10, 15). Viruses can carry genes encoding structural proteins and stress-response functions that help hosts adapt to environmental pressures such as high temperature, acid stress, and heavy metal exposure, thereby influencing microbial evolution and ecosystem resilience (18–20). However, the role of viruses in modulating host responses and ecological strategies to salinity stress remains largely unexplored.

Viral microdiversity, reflecting genetic heterogeneity within populations, is a key determinant of adaptive potential under changing environmental conditions (21, 22). Such diversity is shaped by multiple selective forces, including purifying selection, which removes deleterious mutations, and positive selection, which promotes beneficial adaptations (23). The relative strength of these pressures can vary with environmental context, including host availability, salinity, nutrient levels, and population bottlenecks, which may influence viral mutation rates, genome plasticity, and selection regimes (24, 25). In high-salinity environments, for instance, viruses may experience intensified purifying selection, leading to reduced genetic variability and constrained evolutionary potential, whereas more dynamic or fluctuating habitats may allow greater diversification (26, 27). Despite growing recognition of microdiversity as a key dimension of viral evolution, its environmental drivers remain poorly resolved, particularly across natural estuarine-coastal salinity gradients.

This study employs metagenomic and viromic approaches to comprehensively investigate viral communities across estuarine-coastal regions of China. We aimed to characterize viral diversity and community composition along salinity-driven environmental gradients and explore viral adaptations and evolutionary dynamics to varying salinity conditions. Our findings provide new insights into virus-driven ecological processes by revealing salinity-associated viral adaptations.

## MATERIALS AND METHODS

### Sample collection and extraction

Surface water samples were collected from three typical estuarine-coastal regions in China: the Yangtze River Estuary (YRE, *n* = 4 stations, *n* = 20 samples), the Jiulong River Estuary (JRE, *n* = 4 stations, *n* = 20 samples), and the Pearl River Estuary (PRE, *n* = 5 stations, *n* = 15 samples). The sampled salinity ranges were 0.5–34 PSU in the YRE, 0.2–29 PSU in the JRE, and 0.4–34 PSU in the PRE. Stations were categorized into three salinity groups: low-salinity (0–9 PSU), medium-salinity (10–29 PSU), and high-salinity (>29 PSU), enabling detailed investigation of salinity-driven community dynamics (Table S1; Fig. S1).

Water samples (~10 L) were pre-filtered through 60 µm nylon sieves. Sequential filtration was performed using membranes with pore sizes of 10 µm, 3 µm, 1.2 µm, and 0.2 µm, with viral particles concentrated via iron chloride precipitation and subsequently collected on 0.2 µm membranes (only 3 µm, 0.2 µm, and viral fractions were processed for PRE) (28). *In situ* temperature and salinity were recorded by the CTD sensors during sampling. The concentrations of nitrate, nitrite, and phosphate were measured using the AA3 Auto-Analyzer (AA3, Seal, Germany). The total prokaryotic abundance was analyzed by flow cytometer (Accuri C6, Becton Dickinson, United States).

For each sample, DNA and RNA were extracted simultaneously. The metatranscriptomic data were therefore derived from the same samples used for metagenomic analyses. DNA/RNA was extracted using the DNA/RNA Miniprep Kit (Zymo Research Corporation), according to the manufacturer’s instructions. Sequencing of metagenomes and metatranscriptomes was performed on the Illumina NovaSeq 6000 platform with a paired-end 2 × 150 bp configuration.

### Metagenomic data processing

Quality control of raw reads, assembly of clean reads, and the generation of metagenome-assembled genomes (MAGs) were performed using the MetaWRAP pipeline v1.3.2 (assembly: MEGAHIT; binning: MaxBin2, metaBAT2, and CONCOCT; bin refinement: -c 50 -x 10) (29–33). The bins were then combined and dereplicated using dRep v3.0, applying a 95% average nucleotide identity clustering threshold (34). To assess prokaryotic diversity, phyloFlash was used for the rapid identification and targeted assembly of SSU rRNA from metagenomic data (35).

### vOTU identification, taxonomy, and host prediction

Virus sequences were identified using the ViWrap v1.2.0 pipeline (length threshold: 5000; methods: vb-vs-dvf) (36–39). All identified sequences from the samples were clustered using scripts provided by CheckV v1.0.3 (95% ANI and 85% AF) (40). Following clustering, the representative sequences were assessed for quality using CheckV v1.0.3, and sequences longer than 10 kb or complete were selected for contamination removal. These sequences were then considered as the final vOTU representative sequences.

Viral lifestyles were identified using CheckV v1.0.3 and geNomad 1.7.0 for prophage identification, followed by Bacphlip for prophage induction prediction, along with manual verification of lysogeny-specific genes such as integrase, recombinase, transposase, excisionase, CI/Cro repressor, and parAB (15, 41, 42).

Viral taxonomy was primarily assigned using the marker gene-based classification of geNomad 1.7.0, incorporating BLASTp v2.9.0 against the NCBI RefSeq viral protein database and ICTV VirophageSG results (41, 43, 44). Hosts were predicted using iPHoP v1.3, which infers the host of bacteriophage genomes (45). We used GTDB-Tk 2.3.2 with the de_novo_wf pipeline to infer bacterial and archaeal phylogenetic trees for the estuarine MAGs, which were integrated into the host prediction database (46). The host phylogenetic tree was visualized using tvBOT (47). Additionally, MArVD2 v0.11 was used to distinguish archaeal viruses from bacterial viruses within the viral data set (48).

### Viral diversity and correlation with environmental parameters

To obtain the abundance of virus operational taxonomic units (vOTUs), clean reads from each sample were mapped to the vOTUs using Bowtie2 (49). The generated BAM files, viral vOTU representative sequences, and short-read counts from each metagenome were then used as input for MetaPop to perform diversity analysis (50). Statistical analyses and visualization of diversity and correlation were conducted in R 4.2.2. Diversity indices were calculated using the vegan and ape packages (51, 52). Differences between groups were tested using a *t*-test for normally distributed data or a Wilcoxon test otherwise. To assess the impact of environmental factors on viral abundance and community structure, canonical correspondence analysis (CCA), Spearman correlation, and Mantel tests were performed using the vegan and linkET packages (51, 53). Visualization was conducted using ggplot2 (54).

### Isoelectric points and amino acid frequencies

Protein isoelectric points were calculated for all proteins within selected vOTU proteomes using the *pepstats* function of the EMBOSS package v6.6.0 (55, 56). To determine the isoelectric points and amino acid composition frequencies, the entire proteome of each vOTU was concatenated into a single sequence, and calculations were performed using the pepstats function from the EMBOSS package v6.6.0 (55, 56). Grouped boxplots and heatmaps were generated using ChiPlot (https://www.chiplot.online/).

### Annotation and identification of functional genes

Identification of AMGs and salinity-related genes in viral sequences was performed using DRAM-v v0.1.2 on the KBase platform (auxiliary scores ≤ 3), followed by manual screening (57, 58). In addition, AMGs identified by VIBRANT v1.2.1 were further filtered following established criteria, including removal of edge-located genes, AMGs with KEGG or Pfam v-scores ≥1, AMGs flanked by genes with KEGG v-scores <0.25, and those annotated as COG categories “T” or “B” (59).

### Viral gene abundance and expression

To avoid potential bias from host reads affecting viral gene abundance, vOTU abundance was used to represent the abundance of viral genes, as described above, using MetaPop (50). The expression levels of genes were quantified using Salmon v0.14.1 (60). After quality control of raw reads and removal of rRNA using SortMeRNA v4.3.7, clean transcriptomic reads were used for quantification, and results of expression of genes were reported as transcripts per million (TPM) (61).

### Microdiversity analyses of viral populations

Filtered reads from each metagenomic sample were mapped to representative vOTUs using Bowtie2 (49). The resulting BAM files, along with viral genome assemblies and corresponding read count tables, were processed using the MetaPop pipeline for quality control and microdiversity analysis (50). To ensure reliable single-nucleotide polymorphism (SNP) detection and diversity estimates, only viral populations with an average coverage >10× and >70% genome breadth were retained for downstream analyses (50). Subsampling of SNP frequencies to 10× coverage was performed prior to calculating nucleotide diversity metrics (θ and π) at both the gene and genome levels (62). Additionally, selection pressures on individual viral genes were assessed using the ratio of nonsynonymous to synonymous polymorphisms (pN/pS) and Tajima’s D statistics.

## RESULTS

### Salinity shaped viral community composition despite stable alpha diversity

We identified 137,452 viral sequences, which clustered into 23,163 vOTUs. Viral alpha diversity showed no significant differences across any particle-size fractions or salinity levels (Fig. S2). This pattern contrasts sharply with that of bacteria, whose alpha diversity varied significantly along the salinity gradient (Fig. S3). Nevertheless, salinity significantly structured viral community composition (PERMANOVA, *R*^2^ = 0.147, *P* = 0.001). CCA and Mantel tests indicated that vOTUs correlated with a combination of environmental factors, including temperature, salinity, and their associated environmental factors (Fig. S4).

**Fig 1:**
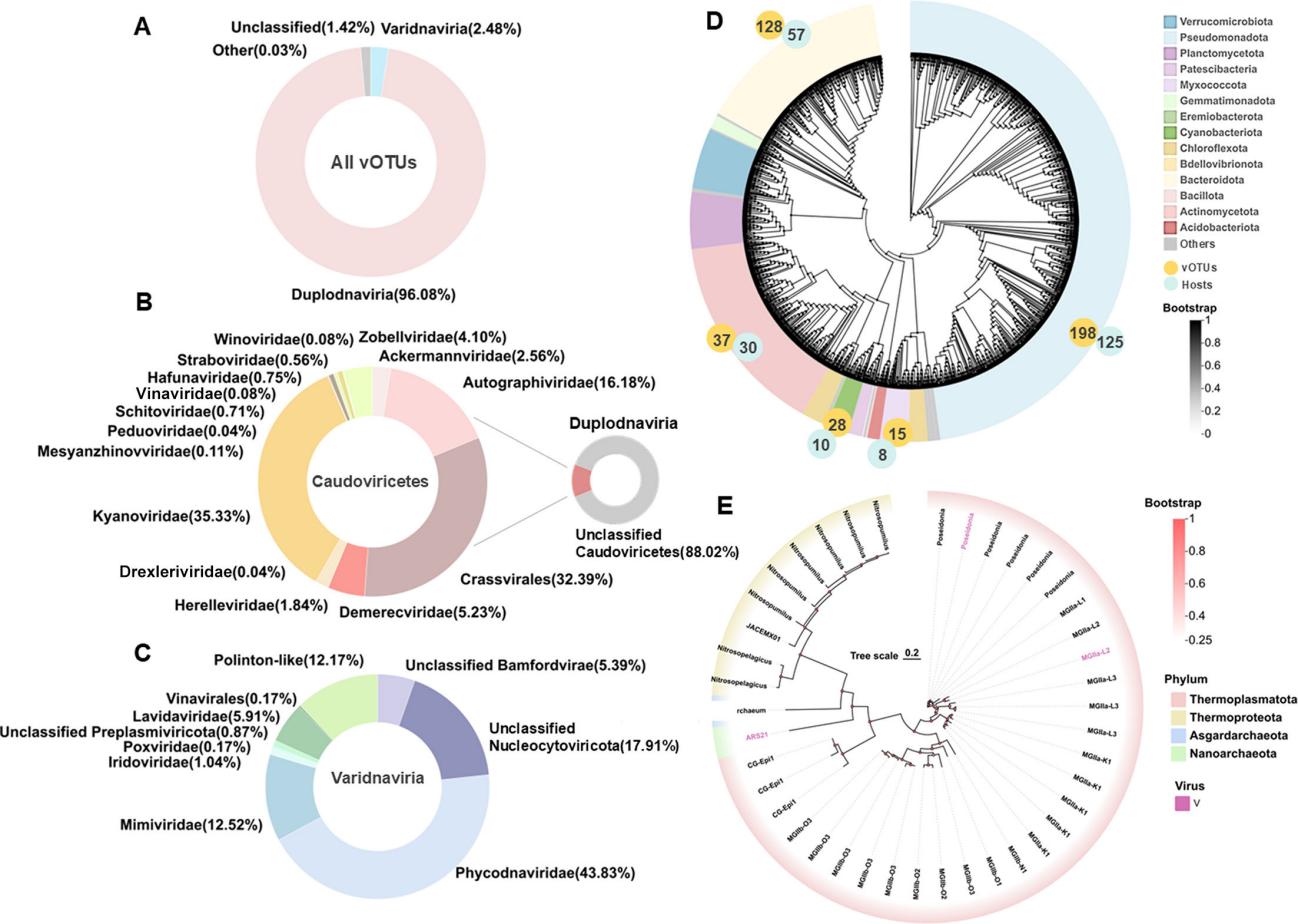
Taxonomic classification and host prediction of vOTUs. (**A**) Realm-level classification of vOTUs. (**B**) Family/order-level classification of Duplodnaviria. (**C**) Family/order-level classification of Varidnaviria. (**D**) Phylogenetic tree of bacterial MAGs and their predicted virus-host associations across the five phyla with the most virus-host links. The tree was reconstructed using GTDB-Tk based on concatenated alignments of conserved single-copy bacterial marker proteins and inferred using a maximum-likelihood approach. Yellow symbols indicate the number of viruses predicted to infect MAGs from each phylum, whereas blue symbols indicate the number of MAGs from each phylum predicted to serve as viral hosts. (**E**) Phylogenetic tree of archaeal MAGs and virus-host associations. Purple symbols indicate archaeal MAGs predicted to serve as viral hosts.

Taxonomic classification (Fig. 1A through C; Table S2) revealed that the majority of vOTUs (*n* = 22,247) were assigned to the class Caudoviricetes, which dominated the estuarine-coastal viral community. Of these, 2,658 vOTUs were further classified into 14 families/orders (Fig. 1B), including Autographiviridae (*n* = 430), Kyanoviridae (*n* = 939), Zobellviridae (*n* = 109), and Crassvirales (*n* = 861). In addition, several vOTUs were associated with viruses infecting eukaryotes (Fig. 1C), including Polinton-like viruses (*n* = 70), Nucleocytoviricota such as Phycodnaviridae (*n* = 252) and Mimiviridae (*n* = 72), and Lavidaviridae (*n* = 34), which are known to co-infect with giant viruses. A small number of single-stranded DNA viruses (Microviridae, *n* = 3) were also detected.

Among these families/orders, eight groups showed significant differences across pore-size fractions, all of which were significantly more abundant in the virome fraction (Fig. S5). Across the salinity gradient, 20 groups exhibited significant variation. Most Caudoviricetes, Phycodnaviridae, and Mimiviridae were enriched under mid- to high-salinity conditions, whereas Corticoviridae and Iridoviridae tended to be more abundant in low-salinity waters. Straboviridae showed higher abundances in mid-salinity environments. This finding highlights the significant role of salinity in shaping viral community structure.

Archaeal viruses also constituted important components of the virome in estuarine-coastal environments; their community composition also differed significantly across salinity groups (PERMANOVA, *R*² = 0.123, *P* = 0.001). A phylogenetic analysis of 129 archaeal virus major capsid protein genes from estuaries revealed novel evolutionary branches within the Magrovirus A group (Fig. S6) (63). Among the predicted archaeal viruses, 118 vOTUs had completeness higher than 50%, and 18 vOTUs were classified as complete sequences (Table S2). These vOTUs were either assigned to the Caudoviricetes or remained unclassified, with most not clustering with known groups in the VipTree database (64). More advanced methodologies are necessary to establish accurate connections between unknown viruses and their respective hosts.

### Prokaryotic diversity and host prediction

SSU rRNA sequences reconstructed from metagenomes revealed that bacterial and archaeal community structures varied distinctly along salinity gradients and across different particle-size fractions (Fig. S3A and B; Table S3). At the class level, archaea exhibited more pronounced distributional differences along salinity gradients, with the relative abundance of Thermoplasmata increasing toward higher salinity, while Nanoarchaeia decreased. Bacterial communities were relatively more stable, although Cyanobacteria showed a higher relative abundance in larger particle-size fractions. Actinobacteria and Verrucomicrobiae gradually decreased in relative abundance from estuary to nearshore environments. In parallel, alpha diversity analysis indicated significantly lower diversity in high-salinity samples compared to other salinity levels (Fig. S3C and D). Beta diversity further revealed substantial community overlap between medium-salinity and both low- and high-salinity zones, indicating active microbial mixing (Fig. S3E).

Furthermore, we reconstructed 1,629 metagenome-assembled genomes (MAGs) at the species level (clustered at 95% ANI), representing 4 archaeal and 27 bacterial phyla. The majority of MAGs belonged to Pseudomonadota (*n* = 781), Actinomycetota (*n* = 243), and Bacteroidota (*n* = 230) (Table S4). A total of 3,921 vOTUs were predicted to have potential hosts, generating 10,725 connections involving 8 archaeal phyla, primarily Nanoarchaeota and Thermoplasmatota, and 37 bacterial phyla, predominantly Pseudomonadota and Bacteroidota. These groups also dominated the microbial community based on SSU rRNA analysis (Fig. S3). By matching with the estuarine-coastal MAGs, 3 vOTUs and 446 vOTUs were predicted to infect *in situ* archaeal and bacterial hosts (Fig. 1D and E; Table S5). Among these, 227 vOTUs were associated with Pseudomonadota. Despite the lower number of recovered Bacteroidota MAGs, a comparable number of vOTUs were predicted to infect Bacteroidota.

### Viral adaptation to salinity through protein-level changes and genes associated with host stress responses

**Fig 2:**
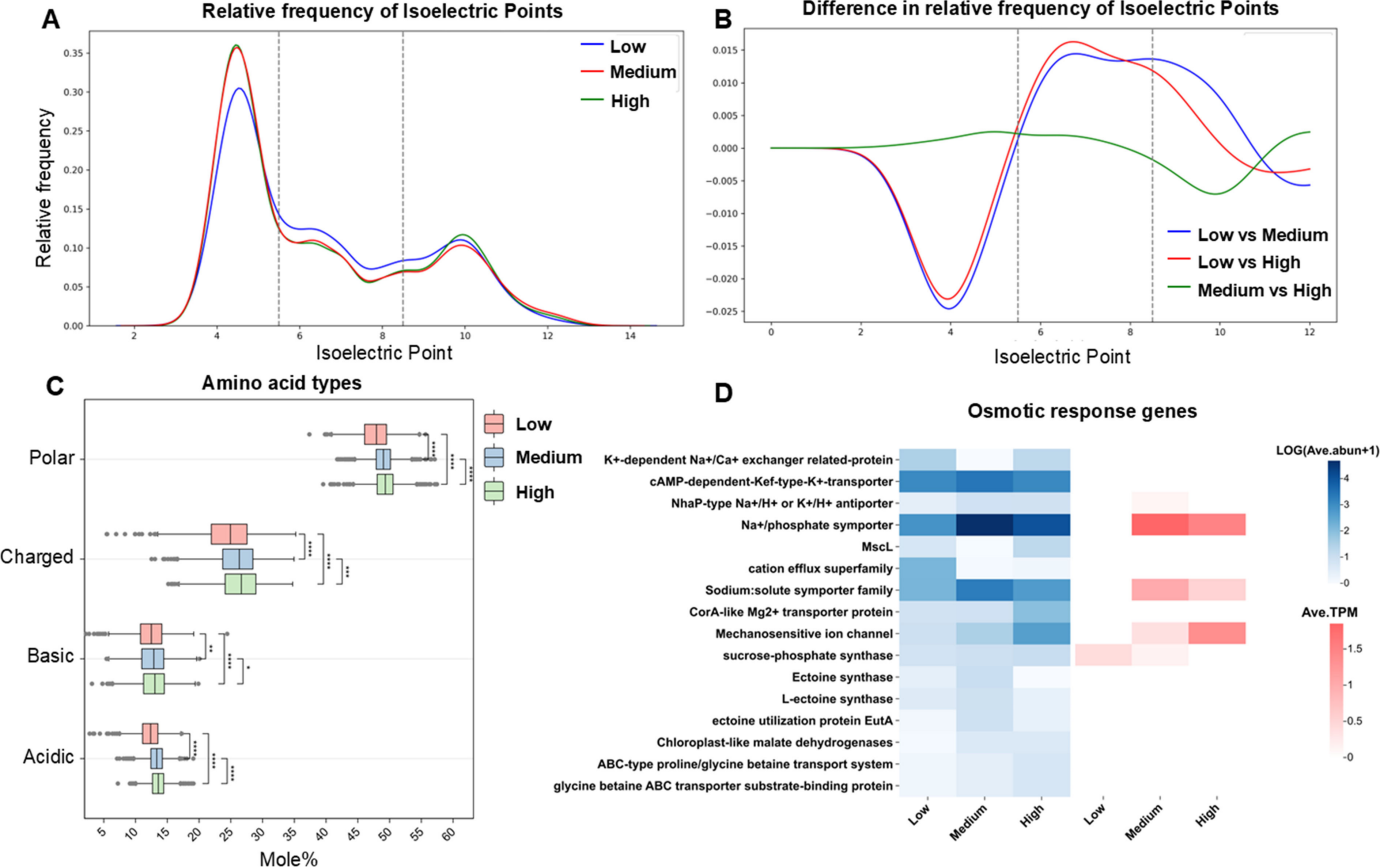
Variation in viral proteome characteristics and salinity-related gene abundance across different salinity groups. (**A**) Average distribution of pIs in viral proteomes. (**B**) Differences in pI frequency distributions among salinity groups. (**C**) Differences in viral protein composition among salinity groups. Statistical significance was assessed using the Wilcoxon test (*P  <  0.05, **P  <  0.01, ***P  <  0.001, ****P  <  0.0001). (**D**) Metagenomic (blue) and metatranscriptomic (red) relative abundances of salinity-related genes across salinity groups.

Although viral alpha diversity did not mirror the distinct salinity gradient differences observed among prokaryotic communities, variations in the distribution of different vOTUs were observed. To further investigate the impact of salinity on viral communities, we compared the average distribution of genome-encoded protein isoelectric points (pIs) among different salinity groups. We focused on viruses that were exclusive to low- or high-salinity metagenomes/viromes, as well as highly enriched in medium-salinity samples (normalized abundance >100-fold relative to the other salinity groups). While the proportion of neutral proteins (pI ∈ [5.5, 8.5]) was elevated and acidic pIs were less frequent in low-salinity environments, only minor differences were observed between the medium- and high-salinity groups (Fig. 2A and B). Additionally, the proportion of polar amino acids, charged amino acids, and basic and acidic amino acids increased broadly with rising salinity (Fig. 2C).

Furthermore, viral sequences carried genes related to osmoregulation. These genes relate to two main microbial strategies for coping with salinity stress: the “salt-in” mechanism, which maintains osmotic regulation via ion balance, and the “salt-out” mechanism, which involves compatible solute uptake or synthesis (5) (Fig. 2D; Table S6). Sodium-related symporters were notably abundant in estuarine-coastal environments, particularly in medium-salinity transitional regions. Genes associated with ectoine exhibited higher abundance in medium-salinity areas, while betaine-related genes were more abundant in high-salinity areas. Interestingly, betaine-related genes showed greater abundance in free-living viral populations (virome) at higher salinity, whereas they were more abundant in metagenomes at medium salinity (Fig. S7). Key components of the “salt-in” strategy were also present in estuarine-coastal viruses. Genes involved in K^+^ and Na^+^ transport, including K^+^ transporter, K^+^(Na^+^)/H^+^ antiporter, and Na^+^/phosphate symporter, were more abundant at medium salinity (Fig. 2D). In contrast, Na^+^/Ca²^+^ exchangers and K^+^ transporters from Caudoviricetes showed an opposite pattern. Genes encoding mechanosensitive channel *MscL* and those associated with Mg^2+^ and Cu^2+^ ion transport increased in relative abundance with rising salinity. These osmoregulation-related genes were mainly from Caudoviricetes, while K^+^ transporters and betaine lipid synthase were found in the giant virus family Phycodnaviridae. Among the 47 vOTUs carrying salinity-related genes, only four were predicted to exhibit a lysogenic lifestyle. Moreover, metatranscripts corresponding to five categories of salinity-related genes were detected, with expression predominantly occurring in the medium- to high-salinity stations.

### Various AMGs involved in biogeochemical cycling in estuarine-coastal viruses

**Fig 3:**
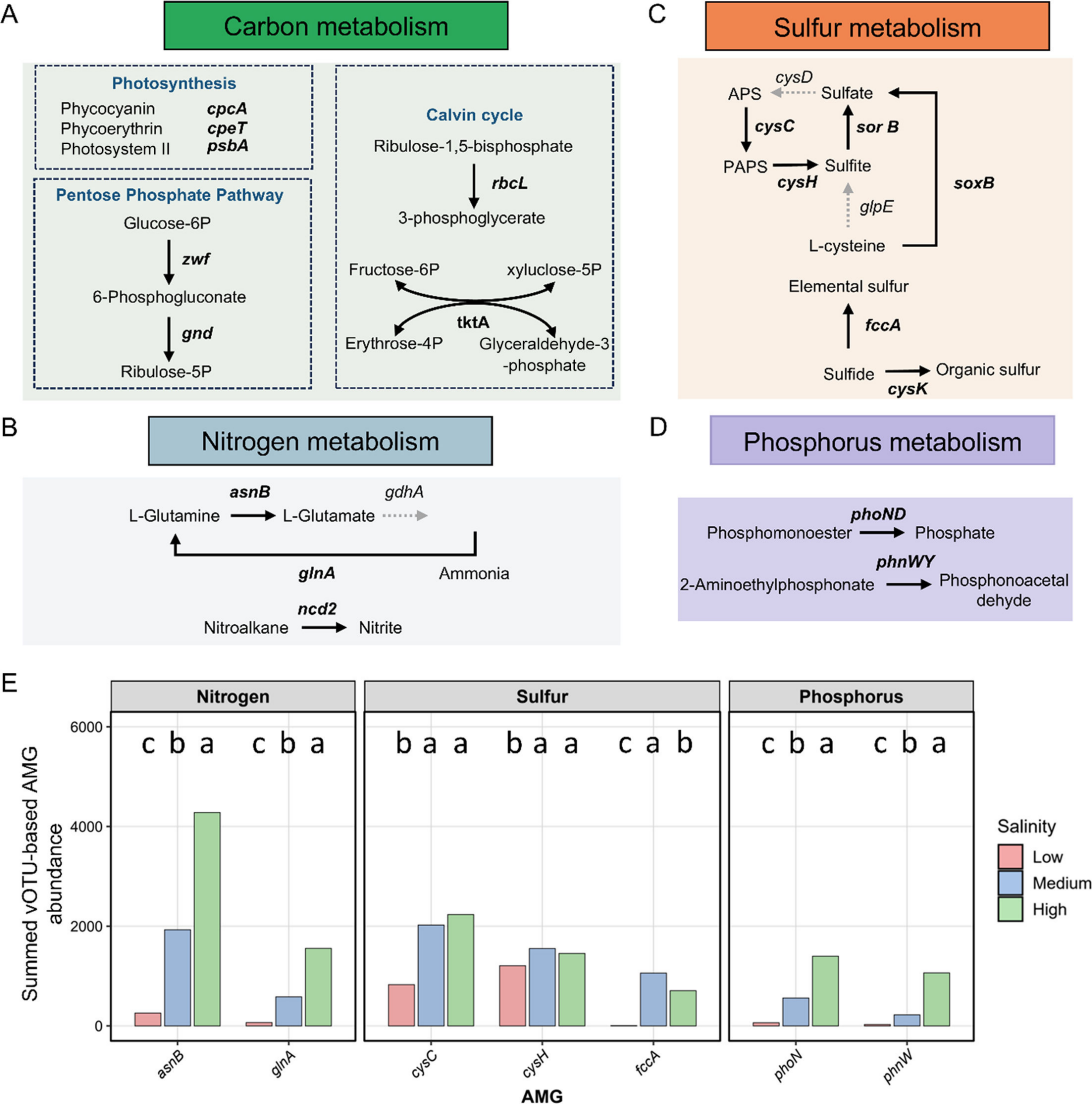
AMGs involved in carbon (**A**), nitrogen (**B**), sulfur (**C**), and phosphorus (**D**) metabolic pathways identified in estuarine-coastal viral genomes. Significantly enriched nitrogen-, sulfur-, and phosphorus-related AMGs across salinity gradients (**E**). Summed vOTU-based abundance of representative nitrogen-, sulfur-, and phosphorus-related AMGs across low-, medium-, and high-salinity groups. AMG abundance was approximated using the abundance of the corresponding AMG carrying vOTUs. Different letters above bars indicate significant differences among salinity groups based on pairwise Wilcoxon tests (*P* < 0.05). APS, adenosine 5'-phosphosulfate; PAPS, 3'-phosphoadenosine 5'-phosphosulfate. Dotted arrows represent steps not encoded by viral AMGs.

We identified a total of 1,922 AMGs from estuarine-coastal viral genomes, clustering into 181 KEGG ortholog groups (KOs). Among these, only 45 AMGs were encoded by lysogenic viruses or prophages. These AMGs encoded diverse functions spanning carbon, nitrogen, sulfur, and phosphorus metabolic pathways, with the potential to reprogram host metabolism and reshape biogeochemical cycling in marine systems (Table S7). In the carbon cycle, viral AMGs were involved in both photosynthetic electron transfer and CO₂ fixation, with genes such as *psbA*, *cpcA*, and *cpeT* linked to light-harvesting processes, whereas *rbcL* and *tktA* were assigned to participation in the Calvin cycle (Fig. 3A). Viral AMGs in the pentose phosphate pathway (e.g., *zwf*, *gnd*) can generate precursors for nucleotide and cofactor biosynthesis, as well as provide reducing equivalents (NADPH) for fatty acid synthesis and deoxyribonucleotide production, suggesting potential roles in host core metabolic pathways. However, host prediction based on *in situ* MAGs revealed matches for only 47 AMGs encoding viruses, indicating the majority lacked identifiable hosts. Among the predicted hosts, Flavobacteriaceae and Woeseiaceae were predominant. The AMGs carried by these host-associated viruses mainly encoded enzymes such as *queE*, *queD*, and *queC*, highlighting viral involvement in queuosine biosynthesis, a process linked to tRNA modification and translational control in hosts.

Nitrogen-related AMGs (*glnA*, *asnB*, *gdhA*) were widespread, with *glnA* and *asnB* exhibiting the highest abundance in high-salinity samples, suggesting potential salinity-driven enrichment (Fig. 3B and E). Additionally, *ncd2* may catalyze the conversion of nitroalkanes to nitrite, a step linking organic nitrogen degradation to the nitrogen cycle. Sulfur metabolism genes, including *cysC*, *cysH*, *sorB*, *soxB*, and *fccA*, were detected in pathways associated with cysteine biosynthesis/breakdown, methionine salvage, sulfonate conversion, and thiosulfate reduction, collectively pointing to sulfide as a central end product (Fig. 3C). Among them, *cysC*, *cysH,* and *fccA* were most abundant in medium- and high-salinity samples (Fig. 3E). Finally, phosphorus-related AMGs (*phoN*, *phpD*, *phnW*, *phnY*) were associated with phosphomonoester and phosphonate utilization (Fig. 3D). Notably, *phoN* and *phnW* showed significantly higher abundance in high-salinity environments (Fig. 3E).

In addition to elemental nutrient cycling, viral AMGs were also involved in nucleotide and cofactor biosynthesis (Fig. S8A). Genes such as *purF*, *purN*, *purM*, and *purA* were associated with *de novo* purine biosynthesis, while *pyrDI* and *pyrF* participated in the pyrimidine biosynthesis pathway. Among these, *purN* and *purC* were most abundant in high-salinity samples, whereas *purA* showed the highest abundance in low-salinity conditions (Fig. S8C). We also detected AMGs involved in folate metabolism, including *folE2*, *queD*, *GCH1*, *phhB*, *moaA*, and *moeZR*, which were assigned to folate-, tetrahydrobiopterin-, and molybdopterin-related pathways (Fig. S8B).

Metatranscriptomic analyses revealed salinity-dependent expression patterns that were broadly consistent with genomic AMG distributions, with medium- and high-salinity regions exhibiting substantially higher transcriptional activity (Fig. S9). Several AMGs enriched at the genomic level, such as *asnB*, *purN*, and *folE2*, also showed elevated expression in higher-salinity waters. The most highly expressed AMGs (e.g., treT, CMAS, SQD1) showed strong salinity-linked upregulation, suggesting potential involvement in host osmolyte metabolism and membrane-related processes, which may influence host-virus interactions in high-salinity environments.

### Viral microdiversity and selection patterns across salinity gradients

**Fig 4:**
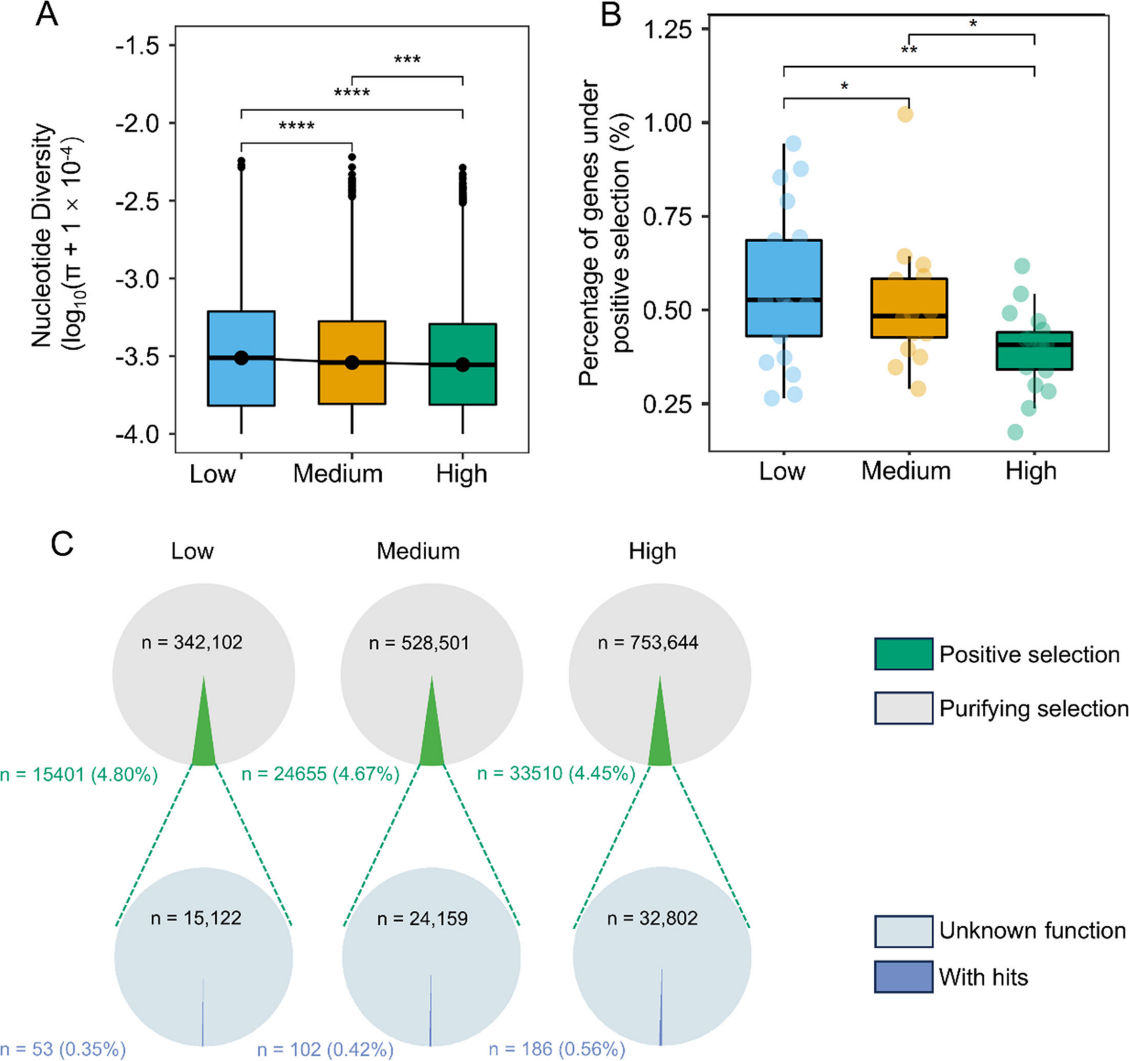
Viral microdiversity and selection patterns across different salinity zones. (**A**) Nucleotide diversity (log_10_-transformed π) of viral contigs in distinct salinity zones. (**B**) Proportion of viral genes under positive selection (pN/pS > 1) in each salinity zone. (**C**) Viral genes classified by selection pressure (pN/pS): positive selection (>1) and purifying selection (<1). Total gene counts (N) and counts for each category are shown per salinity group. Statistical significance is indicated as follows: *P  <  0.05, **P  <  0.01, ***P  <  0.001, ****P  <  0.0001 (Wilcoxon rank-sum test).

To explore viral microdiversity along the estuarine salinity gradient, we assessed nucleotide diversity (π) and gene-level selection pressure. Viral nucleotide diversity ranged from 0 to 3.99  ×  10⁻³, with significantly higher values observed in low-salinity samples, intermediate values in mid-salinity zones, and the lowest diversity in high-salinity waters (Fig. 4A; Fig. S10A). This pattern suggests greater within-population genetic variability in low-salinity environments.

Selection pressure was quantified using pN/pS ratios across viral genes. Overall, 95.7% of viral genes had pN/pS ratios below 1 (mean = 0.022), indicating pervasive purifying selection. Notably, pN/pS values decreased significantly and consistently along the salinity gradient, from low- to mid- to high-salinity samples (mean  =  0.0253, 0.0236, and 0.0204, respectively; Wilcoxon rank-sum test, *P*  <  0.001; Fig. S10B), highlighting intensifying purifying selection in high-salinity environments. We further calculated the proportion of viral genes under positive selection (pN/pS  >  1) in each sample. This proportion declined significantly with increasing salinity, highlighting a salinity-associated gradient in adaptive evolutionary potential (Fig. 4B), and a similar decreasing trend was also observed when genes were pooled by salinity group (Fig. 4C). The majority of positively selected genes were functionally associated with DNA metabolism, CAZymes, and cobalamin synthesis (Fig. S11). More detailed annotation further identified genes involved in folate and one-carbon metabolism, porphyrin and chlorophyll metabolism, and carbohydrate-related pathways (Table S8), indicating that viral genes linked to host metabolic modulation and host-associated interactions may be important components of adaptive evolution in dynamic estuarine environments.

## DISCUSSION

### Salinity shaped prokaryotic and viral communities, whereas viral alpha diversity remained stable

Salinity gradients in estuarine-coastal environments strongly influence microbial community composition and dynamics (4, 6). However, several aspects of viral ecology along salinity gradients remain insufficiently understood, particularly regarding viral functional potential and evolutionary adaptation.

As salinity stress increases during the transition from freshwater to seawater, prokaryotic microbial diversity significantly decreases, accompanied by pronounced shifts in community structure. Pseudomonadota consistently dominate across nearly all salinity levels, whereas Actinobacteriota abundance declines with increasing salinity. This pattern aligns with prior observations in similar ecosystems, where K^+^ uptake has been shown to play a key role in salinity stress adaptation in microbial groups (6). In contrast, archaeal communities show more pronounced structural shifts at the class level across the salinity gradient, indicating a potentially narrower tolerance range for salinity and higher sensitivity to specific salinity conditions (65).

Given the potential host background described above, we hypothesized that viruses also exhibited diversity variation along salinity gradients, adapting either directly to salinity stress or indirectly by assisting host adaptation. Most viral taxa showed significant variation along the salinity gradient. However, viral alpha diversity showed relatively stable patterns without significant shifts along the gradient at the vOTU level. Similar patterns have been observed in the Delaware and Chesapeake Bays (66). One possible explanation is that viruses may possess higher tolerance to fluctuations in ionic strength compared to their host cells (67). In addition, higher viral genomic diversity and contributions from both prokaryotic and eukaryotic hosts may also contribute to this pattern (68).

Notably, we showed the viral and microbial community variation across salinity categories. Although salinity represents one of the major environmental gradients in estuarine systems, community variation is also influenced by multiple covarying factors, such as temperature, nutrient availability, turbidity, and geographic differences.

### Viral protein properties mirror bacterial patterns, and vOTUs carry salinity adaptation genes

Microorganisms typically display distinct amino acid composition between marine and freshwater habitats, reflecting differences in proteome pIs (12, 56). Our study demonstrates that estuarine-coastal viruses enriched in medium- to high-salinity regions exhibited more acidic proteomes compared to those exclusive to low-salinity regions. Remarkably, the higher acidity observed in viral proteomes in marine environments mirrors patterns observed in prokaryotic proteomes, although the precise physiological explanation remains unclear (12, 56). Furthermore, viruses enriched in higher-salinity groups exhibit proteomes with significantly increased proportions of polar, charged, basic, and acidic amino acids, consistent with patterns observed in aquatic bacteria (56). These findings suggest that proteomic boundaries between freshwater and saline environments may extend to viral communities.

Acidic amino acids in marine microbes, which may prevent protein aggregation and facilitate interactions with ions such as K^+^, support functional stability in saline conditions (69). Viral proteins must function within the intracellular environment of their hosts. Proteins enriched in acidic residues may enhance solubility and reduce aggregation under such conditions, resulting in a shift toward lower proteome isoelectric points (12). These shifts may also reflect adaptation to local physicochemical conditions associated with salinity, as viral pI influences virion surface charge and may affect adsorption to environmental particles (70).

Furthermore, in estuarine-coastal viral genomes, we identified genes associated with both the “salt-in” and “salt-out” strategies employed by bacteria to cope with salinity stress. The salt-in strategy is often associated with the influx and efflux of ions. Viral sequences enriched in mid-salinity regions frequently contain genes involved in Na^+^-related regulation, a crucial adaptation mechanism for maintaining cellular homeostasis in saline environments (71). Genes encoding transport proteins, such as the solute/sodium symporter family and K^+^ transporters, further illustrate how viral sequences may help host tolerance to osmotic stress (6, 72). Notably, giant virus genomes include K^+^ transporter genes, which may also facilitate their DNA genome injection into host cells (73). Viral sequences also contained genes encoding bacterial homologs of ion channels and transporters (e.g., Mg^2+^ transporter, *CorA*, mechanosensitive ion channels), whose abundances correlate positively with salinity, suggesting potential roles in host osmotic stress management.

A universal microbial strategy for coping with salinity stress involves synthesizing or accumulating organic osmolytes such as sucrose, betaine, and ectoine (5). Betaine biosynthesis in bacteria predominantly occurs in photoautotrophs such as cyanobacteria, although some heterotrophic bacteria generally cannot synthesize betaine but possess uptake systems for it (74, 75). Additionally, bacteria, including Proteobacteria, Firmicutes, and Actinobacteria, along with certain archaea and eukaryotes, can utilize ectoine and its derivatives for osmotic regulation (76). Viral genomes in estuarine-coastal environments harbor genes associated with osmolyte metabolism, suggesting a potential role in host osmotic regulation. Specifically, ectoine-related genes were most prevalent in mid-salinity environments. Viruses containing betaine-related genes show relatively higher abundances in mid-salinity regions in metagenomes, which mainly represent intracellular viral sequences (10–0.2 µm size fraction). However, these viruses are more abundant in viromes from high-salinity regions, representing free-living viral sequences. One possible explanation is that this pattern may reflect shifts in viral lifestyle along the salinity gradient. Viral sequences detected in metagenomes may include integrated prophages associated with lysogenic infections, whereas viromes primarily represent free viral particles produced during lytic cycles. The increased representation of betaine-related genes in these viral sequences at higher salinity may therefore suggest a higher prevalence of lytic viral activity under high-salinity conditions.

Notably, although metatranscriptional evidence was detected for some of these genes, their functional roles in viral genomes remain to be experimentally validated, and their contributions to host adaptation should therefore be considered as potential.

### Salinity constraints on viral microdiversity and evolutionary dynamics

Viral microdiversity has been reported to decrease from freshwater to marine environments, with average nucleotide diversity values of 2.95  ×  10^−3^ in freshwater systems and 3.78  ×  10^−4^ in global ocean viromes (21, 59). However, the estuarine-coastal interface remains poorly characterized in terms of within-population viral genetic variation, despite its steep environmental gradients and ecological complexity (9). In our study, nucleotide diversity decreased significantly along the salinity gradient, highlighting a transitional pattern between freshwater and marine systems. This trend illustrates a gradual continuity of viral microdiversity between freshwater and marine environments, and suggests that salinity exerts a strong and continuous filtering effect on viral population diversification (77).

Elevated nucleotide diversity in low-salinity waters likely reflects higher viral turnover and standing genetic variation, consistent with more dynamic microbial communities and fluctuating host availability (78). In contrast, reduced diversity in high-salinity zones may reflect genetically streamlined viral populations under more stable but physiochemically stressful conditions. These observations point to salinity as a key abiotic driver modulating viral evolutionary potential through its control over community assembly, infection dynamics, and mutation retention (26). Consistently, pN/pS ratios decreased significantly with increasing salinity, indicating a shift toward intensified purifying selection in more saline conditions. At the sample level, the proportion of genes under positive selection also declined with salinity, suggesting that high-salinity viral communities may be under stronger functional constraints and reinforcing the notion that viral evolutionary potential is increasingly limited along the gradient (27, 79).

Despite the dominance of purifying selection across all salinity zones, a small fraction of viral genes (~0.5%) consistently exhibited signatures of positive selection. These genes were primarily associated with functional modules involved in folate and one-carbon metabolism (e.g., *queE*, *glyA*), porphyrin and chlorophyll metabolism (e.g., *ahbD*, *bchE*), and carbohydrate-related pathways (e.g., *galE*, *gmhC*), suggesting that viral functions linked to host metabolic reprogramming and host-associated interactions retain greater evolutionary flexibility in dynamic estuarine environments. Their higher representation in low-salinity samples further indicates that these habitats impose more heterogeneous and fluctuating selective pressures, thereby promoting episodic virus-host co-adaptation (26, 80). By contrast, the increase in purifying selection with salinity likely reflects a narrower range of viable viral genotypes under high-salinity conditions, where stronger physicochemical constraints favor the persistence of optimized variants and more effectively purge deleterious mutations (78, 81). Reduced effective population sizes or recurrent bottlenecks may also contribute to the loss of standing genetic variation in these habitats (82, 83). Collectively, these results highlight salinity as a major selective force shaping viral genetic variability and evolutionary trajectories across estuarine gradients.

### Viral AMGs and biogeochemical cycling in estuarine-coastal ecosystems

The broad repertoire of AMGs identified in estuarine-coastal viral genomes suggests that viruses have the genomic potential to influence host metabolic processes and marine biogeochemical cycling (84). These genes span key functional categories, including carbon, nitrogen, sulfur, phosphorus, nucleotide, and folate metabolism, reflecting the potential for broad metabolic augmentation during infection (14). The presence of such AMGs across all salinity levels suggests a conserved ecological function of viruses in sustaining microbial metabolic capacity in dynamic coastal systems (85).

Notably, the distribution of several AMGs exhibited clear and significant salinity-associated patterns, implying that environmental gradients may act as selective filters influencing the retention or enrichment of specific viral functions (84). Several nitrogen-related AMGs, particularly *glnA* and *asnB*, were enriched in high-salinity environments, suggesting that viruses may support host nitrogen metabolism in outer estuarine zones where elevated salinity coincides with reduced nitrogen availability (86). *GlnA* encodes glutamine synthetase, a key enzyme for ammonia assimilation and amino acid biosynthesis, while *asnB* contributes to asparagine production, both of which may support viral replication under nitrogen stress (17). Likewise, sulfur-related AMGs (*cysH*, *fccA*) peaked in medium- to high-salinity samples; these genes participate in sulfate assimilation and sulfide production, potentially modulating host redox status and stress tolerance during infection (87). Phosphorus-related AMGs (*phoN, phnW*) were also more abundant under high-salinity conditions, highlighting virus-mediated enhancement of phosphorus acquisition in areas where salinity increases and inorganic phosphate declines (88).

Beyond elemental metabolism, viruses may also influence host nucleotide and cofactor pools. Purine biosynthesis genes and folate pathway genes also showed salinity-associated enrichment, particularly under high-salinity conditions (89). These genes support nucleic acid synthesis and redox cofactor production, both of which are essential for viral genome replication and host metabolic rewiring (59, 90). Interestingly, *purA* was the only AMG significantly enriched at low salinity, suggesting that purine salvage pathways may play a distinct role in freshwater-influenced microbial hosts (91).

Importantly, metatranscriptomic data revealed that many of these salinity-enriched AMGs, such as *asnB*, *purN*, and *folE2,* also showed elevated expression in medium- and high-salinity waters, indicating that these functions are not only genomically encoded but actively transcribed under osmotic stress (13). Moreover, the highest expressed AMGs (e.g., *treT*, *CMAS*, *SQD1*) exhibited strong upregulation at higher salinity, suggesting potential involvement in viral modulation of host osmolyte production and membrane remodeling in more saline environments (10).

Altogether, these results underscore the metabolic versatility of estuarine-coastal viral communities and highlight salinity as a key environmental filter shaping the scope and context of virus-mediated metabolic augmentation.

While metagenomics provides powerful insights into the potential functions of viral communities, it primarily offers limited evidence. The actual functional roles of viral genes require experimental validation through wet-lab approaches. In addition, the classic question of “who infects whom” remains difficult to resolve with current approaches, which constrains our understanding of horizontal gene transfer and viral ecological functions. Addressing these gaps will require the integration of multiple approaches, such as MetaHiC, single-cell methods, and experimental validation.

### Conclusions

**Fig 5:**
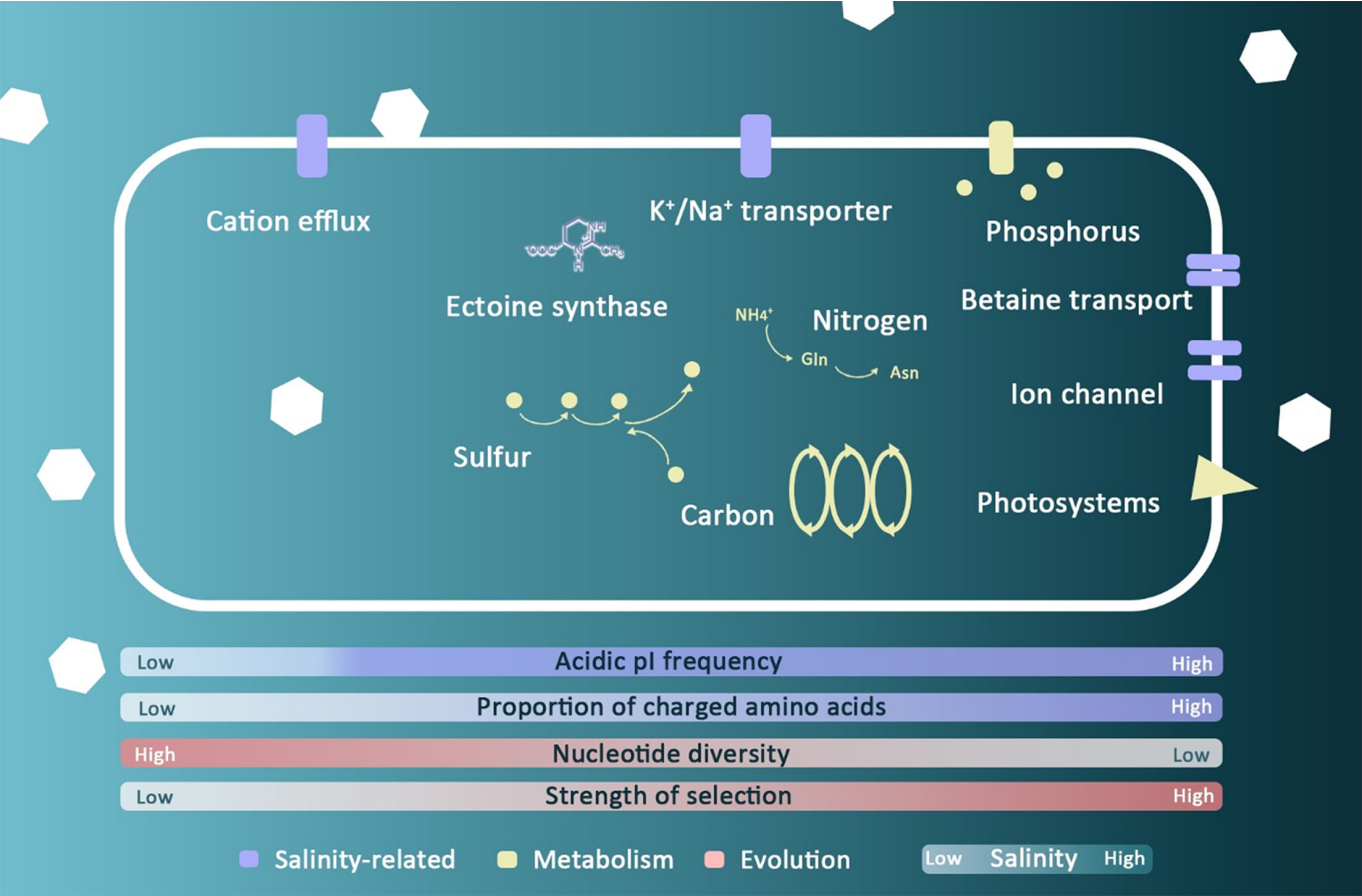
Schematic summary of salinity-associated features and potential functions of estuarine-coastal viruses. Purple indicates salinity-related features and potential salinity-related genes, yellow indicates the potential functions of AMGs, and red indicates evolutionary features of viral communities.

Our study highlights the complex and multifaceted roles of viruses in estuarine-coastal ecosystems, emphasizing their unique adaptations to salinity-driven environmental gradients and their integral contributions to ecosystem functioning (Fig. 5). Through detailed metagenomic and viromic analyses, we identified salinity-associated patterns in viral proteomes and gene content, including shifts in protein isoelectric point and amino acid composition as well as the presence of salinity-related genes, suggesting potential viral responses to changing salinity conditions. In parallel, viral microdiversity declined significantly along the salinity gradient, with reduced nucleotide diversity and pN/pS ratios indicating intensified purifying selection and constrained evolutionary potential in more saline waters. The detection of AMGs further underscores the active role of viruses in host metabolic reprogramming and key biogeochemical processes. Future research should aim to mechanistically link viral functions to ecosystem processes under accelerating environmental change.

## Data Availability

Sequencing data from this study are available in the NCBI Sequence Read Archive (SRA) under the BioProject accession number PRJNA1373522 (SRA accession numbers SRR36426603–SRR36426657). The genomes generated in this study have been deposited in the National Omics Data Encyclopedia (NODE, https://www.biosino.org) under the same project (OEP00004009), with analysis accession number OEZ00021752. Scripts used in this study are publicly available at https://github.com/Wenqing-S/Salinity-driven-adaptations-and-evolution-of-DNA-viruses-in-estuarine-coastal-ecosystems/.

## SUPPLEMENTAL MATERIAL

Supplemental figures msystems.00354-26-s0001.docx Fig. S1 to S11.

Supplemental tables msystems.00354-26-s0002.xlsx Tables S1 to S8.

ASM does not own the copyrights to Supplemental Material that may be linked to, or accessed through, an article. The authors have granted ASM a non-exclusive, world-wide license to publish the Supplemental Material files. Please contact the corresponding author directly for reuse.
