# Supplementary material for: Salinity-driven adaptations and evolution of DNA viruses in estuarine-coastal ecosystems
Source: mSystems. 2026 May 18;11(6):e00354-26. doi: 10.1128/msystems.00354-26 (PMC13288924; doi:10.1128/msystems.00354-26)
Supplement: Supplemental figures — Fig. S1 to S11. [file msystems.00354-26-s0001.docx]

Supplementary Information for

**Salinity-driven adaptations and evolution of DNA viruses in estuarine-coastal ecosystems**

Wenqing Shi^1,2,3^‡, Lu Liu^1,2^‡, Lilin Wu^1,2^, Xiaomeng Wang^1,2,4^, Yongyi Peng^5,6^, Xinyue Liu^5^, Chengpeng Li^5,7^, Jinxin Xu^1,2^, Ziqi Wu^1,2^, Xiyang Dong^5^*, and Qiang Zheng^1,2^*

^1^State Key Laboratory of Marine Environmental Science, Institute of Marine Microbes and Ecospheres, College of Ocean and Earth Sciences, Xiamen University, Xiamen, 361102, China.

^2^Fujian Key Laboratory of Marine Carbon Sequestration, Xiamen University, Xiamen, 361102, China.

^3^ RD3 Marine Ecology, RU Marine Symbioses, GEOMAR Helmholtz Centre for Ocean Research Kiel, Kiel, 24148, Germany.

^4^Department of Ocean Science and Engineering, Southern University of Science and Technology, Shenzhen, 518055, China.

^5^Key Laboratory of Marine Genetic Resources, Third Institute of Oceanography, Ministry of Natural Resources, Xiamen, 361005, China.

^6^Department of Microbiology, Biomedicine Discovery Institute, Monash University, Clayton, VIC 3800, Australia.

^7^Key Laboratory of Advanced Marine Materials, Key Laboratory of Marine Environmental Corrosion and Bio-fouling, Institute of Oceanology, Chinese Academy of Sciences, Qingdao, 266071, China.

‡ These authors contributed equally to this work.

* Correspondence can be addressed to Qiang Zheng ([zhengqiang@xmu.edu.cn](mailto:zhengqiang@xmu.edu.cn)) or Xiyang Dong ([dongxiyang@tio.org.cn](mailto:dongxiyang@tio.org.cn)).

**Contents:**

**Figure. S1** Sampling stations and information for three representative estuarine-coastal areas in China.

**Figure. S2** Viral community Shannon diversity across particle-size fractions (A) and salinity groups (B).

**Figure. S3** Prokaryotic community structure and diversity based on metagenomic recovery of SSU rRNAs.

**Figure. S4** CCA analysis (A) and Mantel analysis (B) of vOTUs and environmental factors.

**Figure. S5** Normalized abundance of vOTUs across samples by classification.

**Figure. S6** Phylogenetic tree of archaeal MCP genes from vOTUs and reference sequences.

**Figure. S7** Abundance of salinity-related genes across Viromes/Metagenomes salinity groups.

**Figure. S8** AMGs involved in nucleotide (A) and folate (B) metabolism identified in viral genomes.

**Figure S9** Heatmap showing significantly differentially expressed viral AMGs across low-, medium-, and high-salinity regions based on metatranscriptomic data.

**Figure S10** Gene-level selection pressure indicated by pN/pS ratios of viral genes across salinity zones.

**Figure S11** Functional classification of viral genes under positive selection (i.e., pN/pS ≥ 1) across salinity gradients.

**Figure S1**


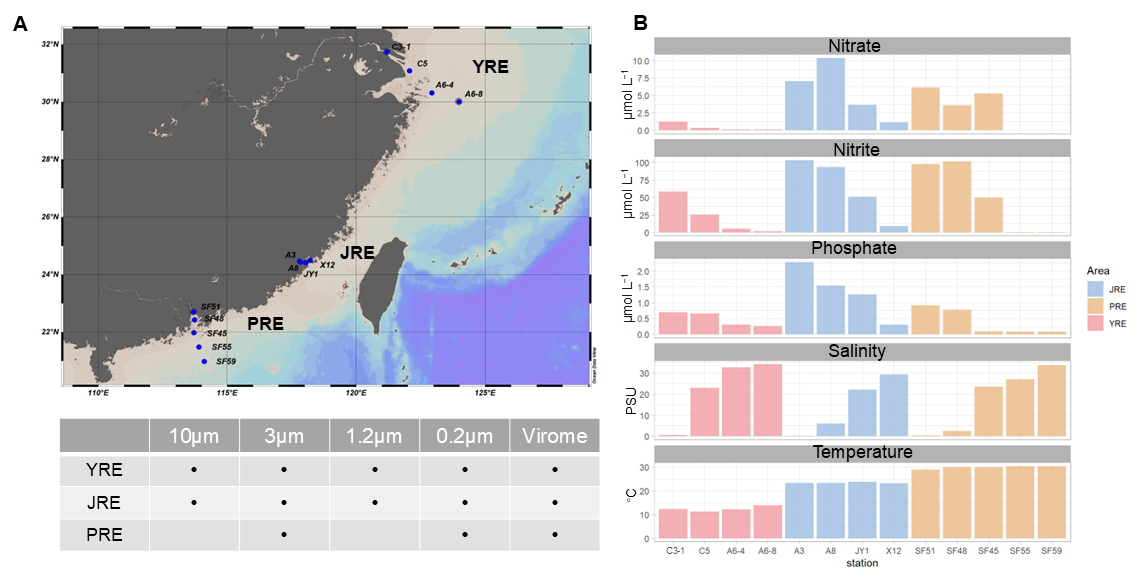


**Figure. S1** Sampling stations and information for three representative estuarine-coastal areas in China. (A) Sampling stations and filter particle size at each station. This image was generated using Ocean Data View software (Schlitzer, R., Ocean Data View, odv.awi.de, 2023). (B) Temperature, salinity, and nutrient parameters for each station.

**Figure S2**

**
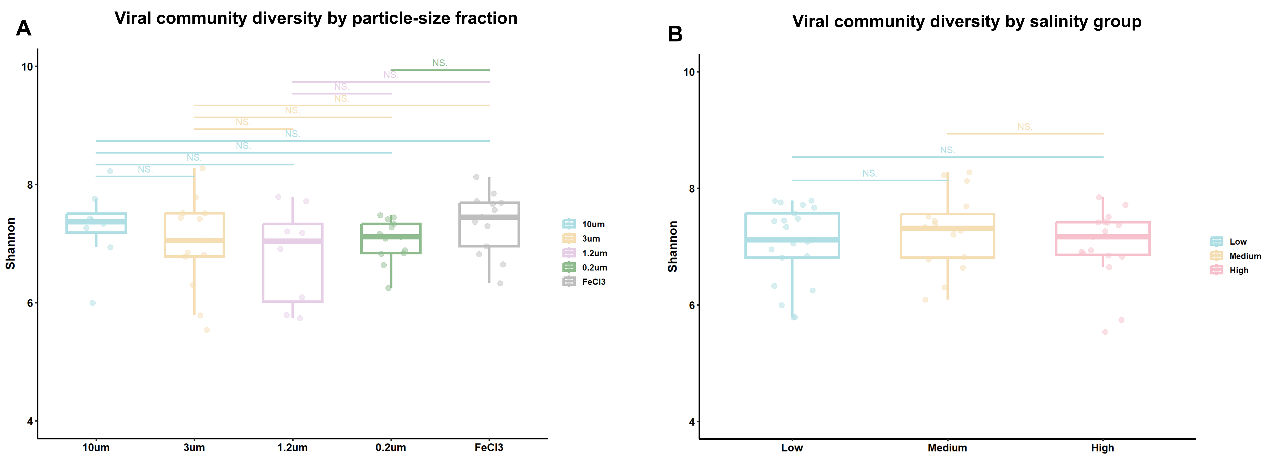
**

**Figure. S2** Viral community Shannon diversity across particle-size fractions (A) and salinity groups (B).

**Figure S3**


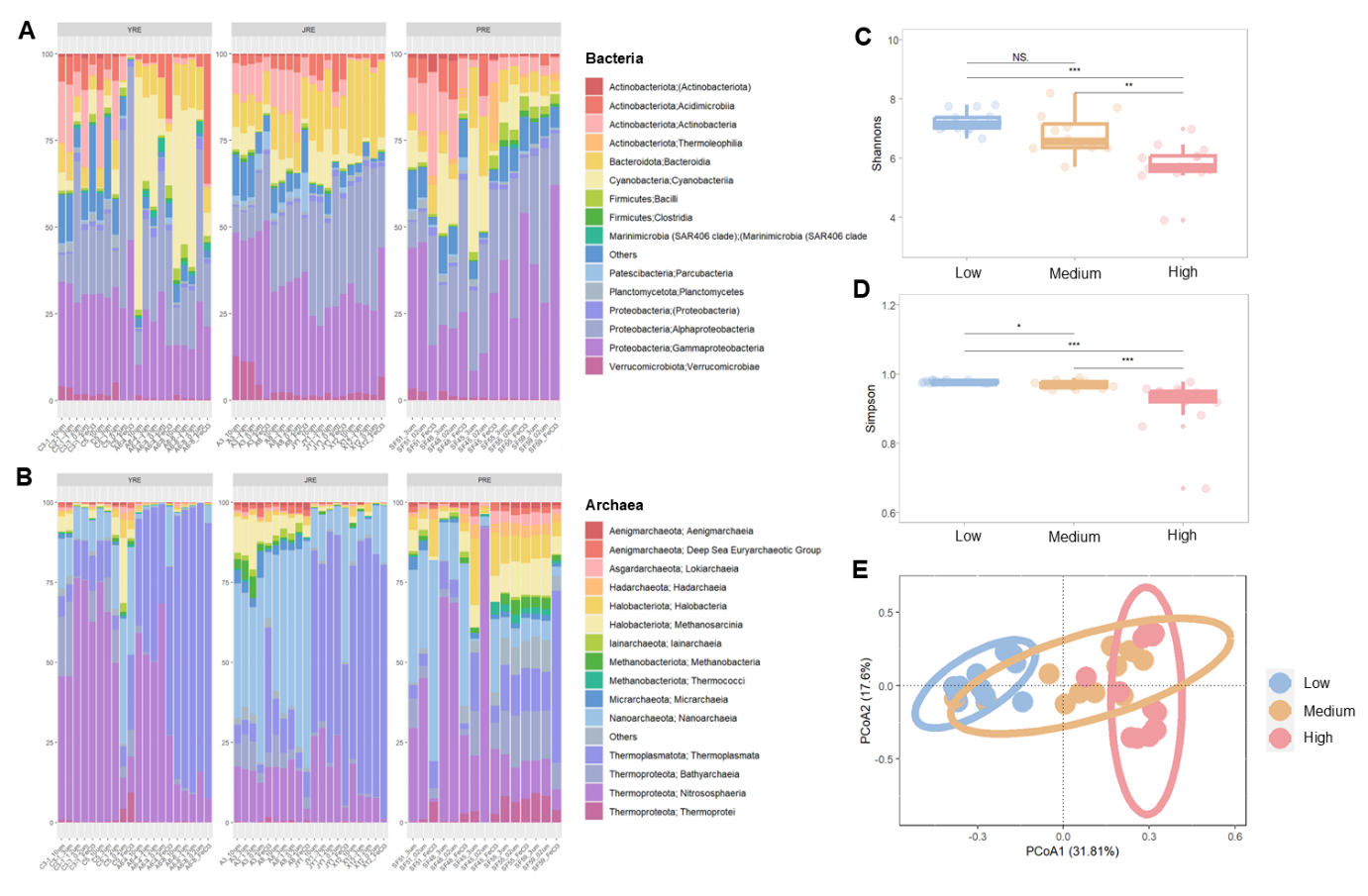


**Figure. S3** Prokaryotic community structure and diversity based on metagenomic recovery of SSU rRNAs. (A) Bacterial community structure of each sample. Particle sizes are arranged from largest to smallest, and sampling sites are ordered according to salinity, from low to high. (B) Archaeal community structure of each sample. (C-D) Shannon and Simpson diversity indices of prokaryotes in each salinity group. One asterisk denotes significance at the 5% level, two asterisks indicate significance at the 1% level, and three asterisks represent high significance. (E) Bray-Curtis dissimilarity calculated from normalized abundances of prokaryotic SSU rRNA, with a 95% confidence ellipse for each salinity group (PERMANOVA, R² = 0.293, *p* = 0.001).

**Figure S4**


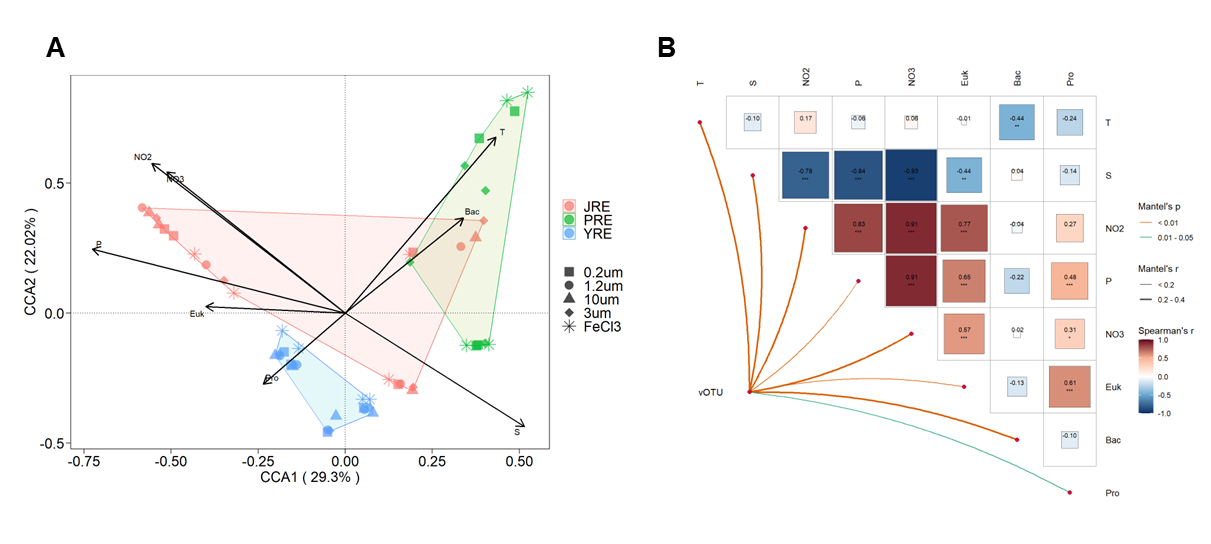


**Figure. S4** CCA analysis (A) and Mantel analysis (B) of vOTUs and environmental factors.

**Figure S5**


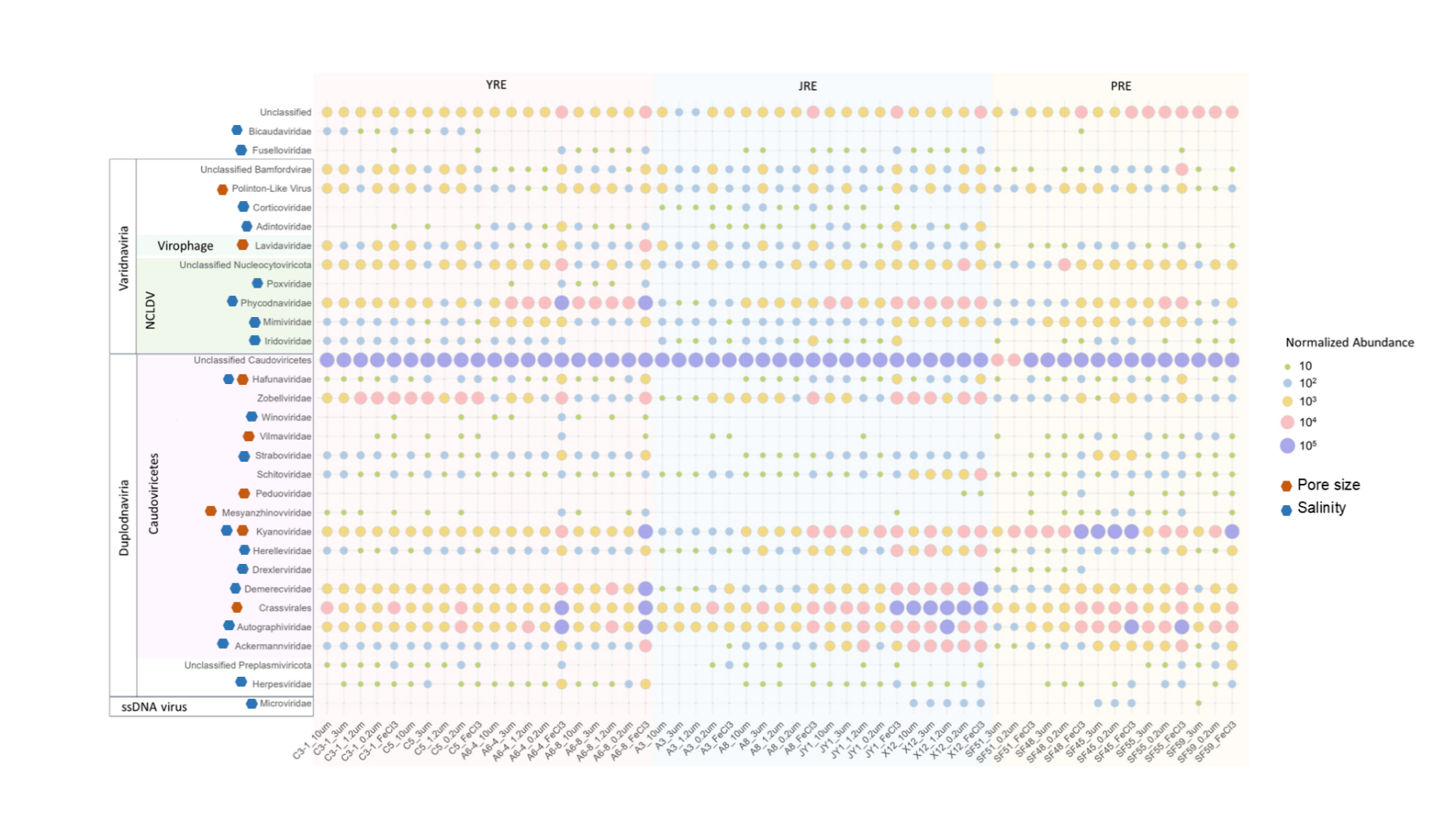


**Figure. S5** Normalized abundance of vOTUs across samples by classification. Red markers indicate that the abundance of this category differs significantly among particle‐size fractions, whereas blue markers indicate significant differences among salinity groups.

**Figure S6**


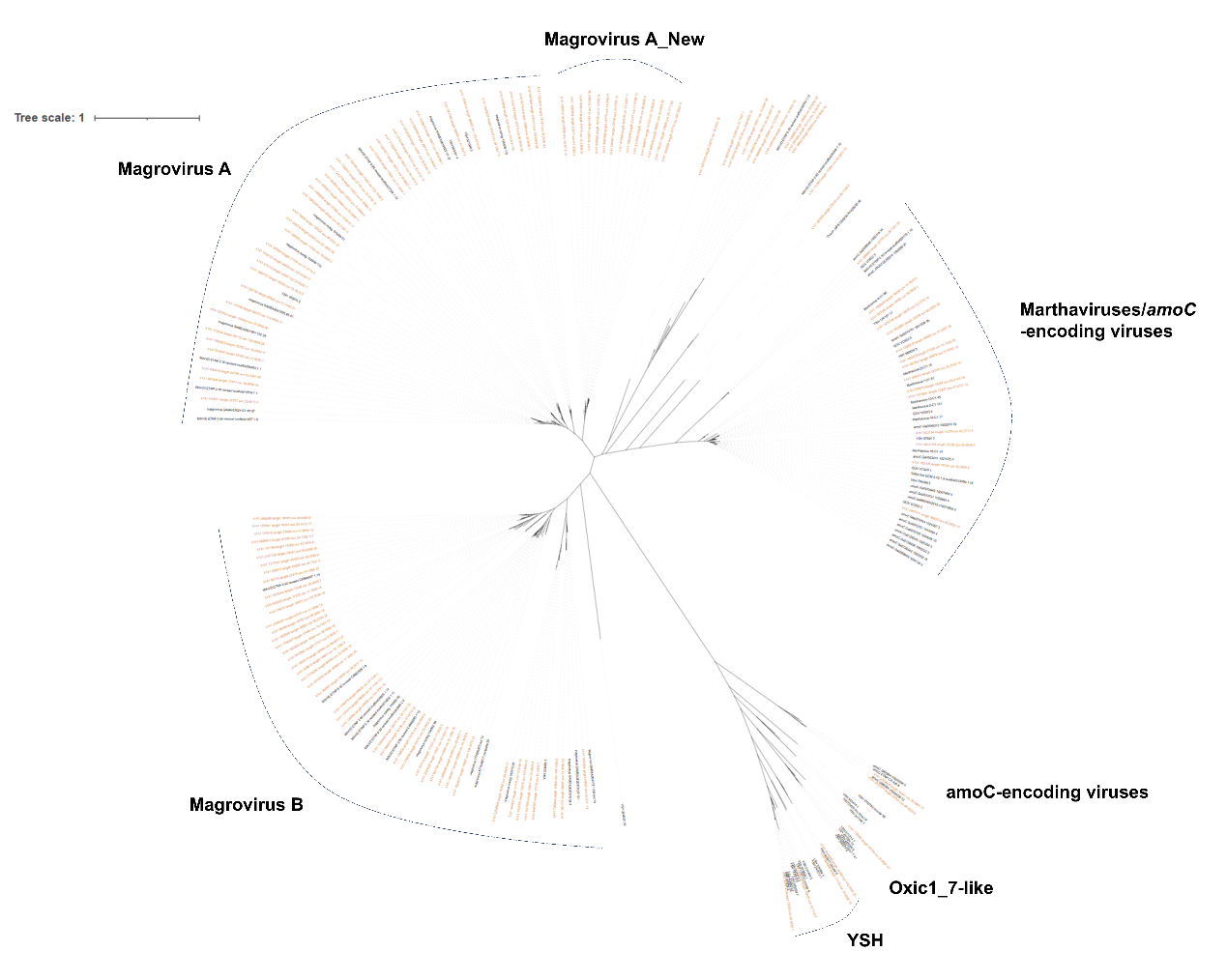


**Figure. S6** Phylogenetic tree of archaeal major capsid protein genes from vOTUs and reference sequences. Orange represents vOTU sequences.

**Figure S7**


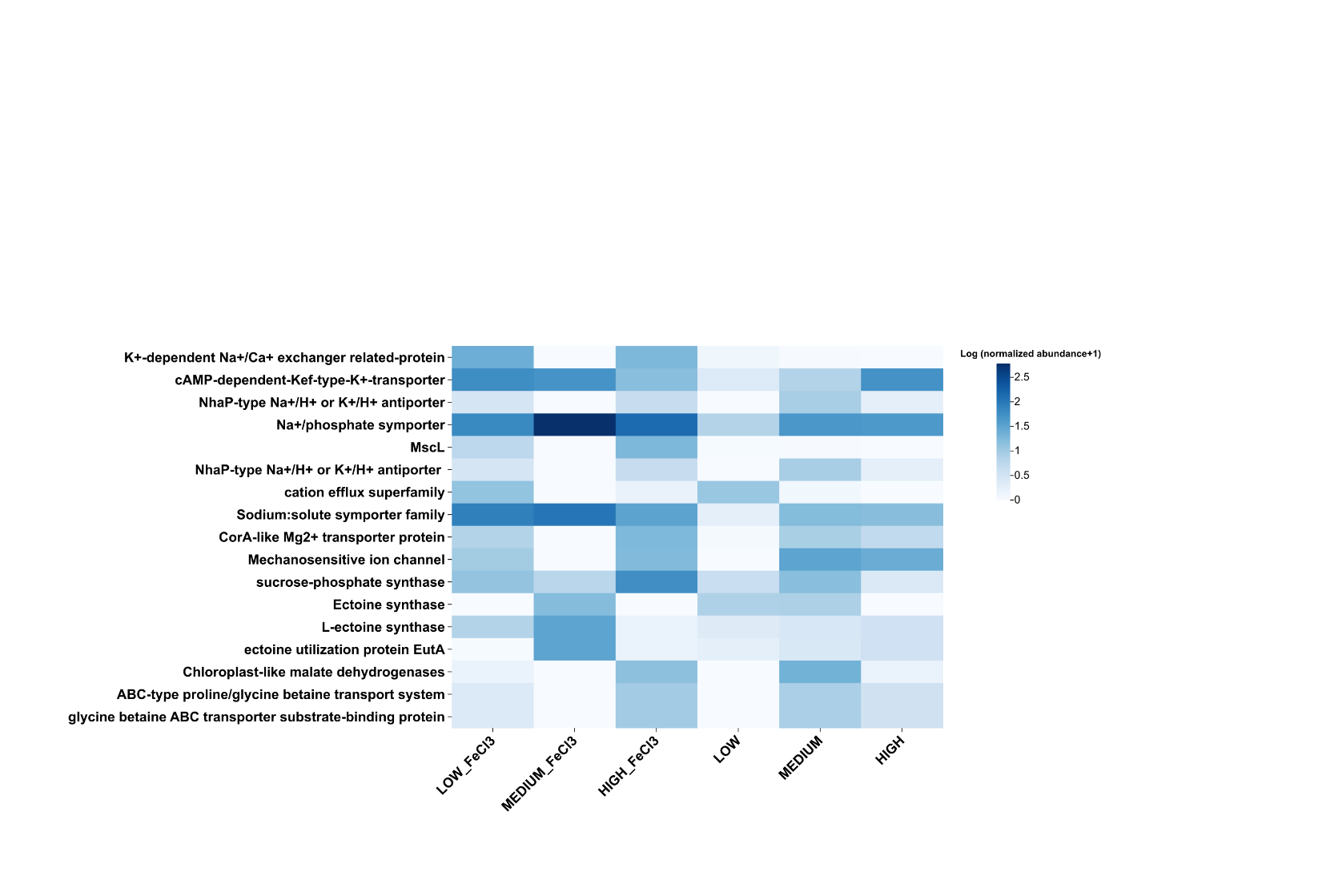


**Figure. S7** Abundance of salinity-related genes across Viromes/Metagenomes salinity groups. The heatmap shows gene abundance with values presented as log (abundance + 1).

**Figure. S8**

**
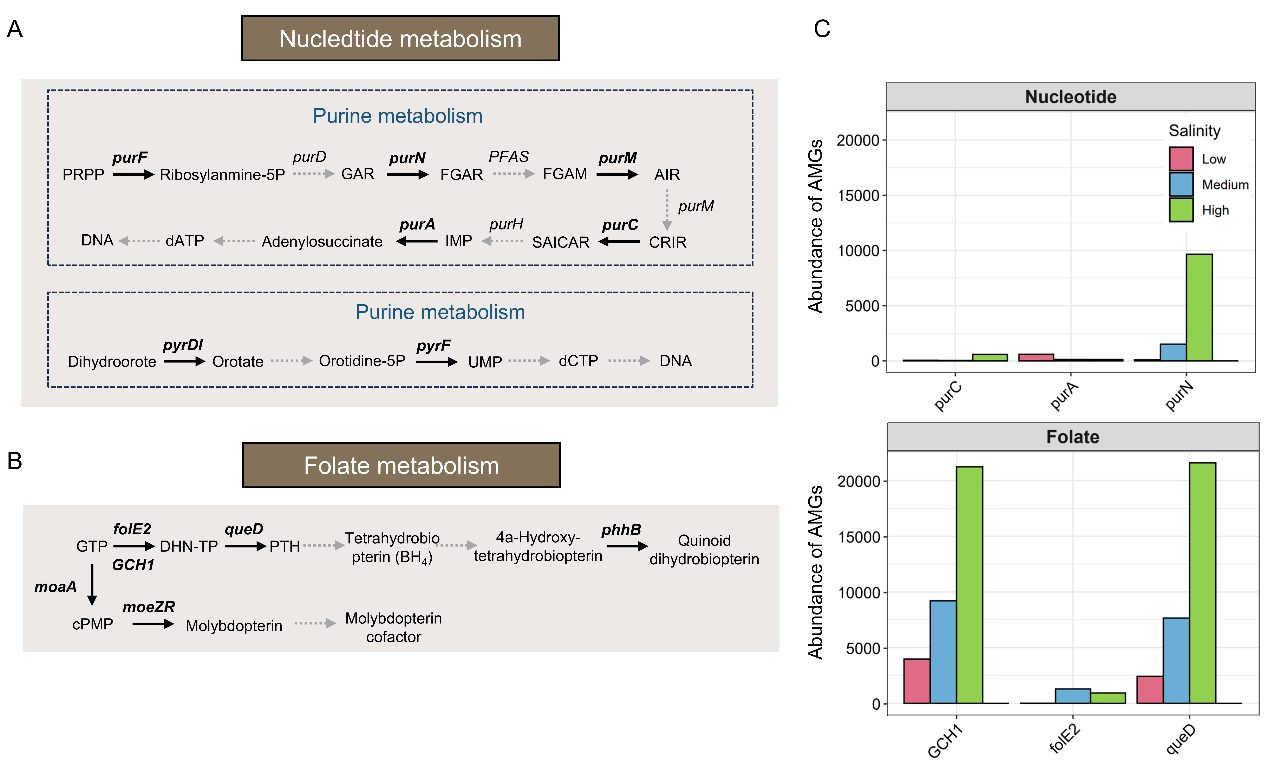
**

**Figure S8.** AMGs involved in nucleotide (A) and folate (B) metabolism were identified in viral genomes. Differential abundance of selected nucleotide and folate-related AMGs across salinity regimes. Bars represent AMGs with statistically significant differences (e.g., *p* < 0.05) among low- (red), medium- (blue), and high-salinity (green) samples.

**Figure S9**


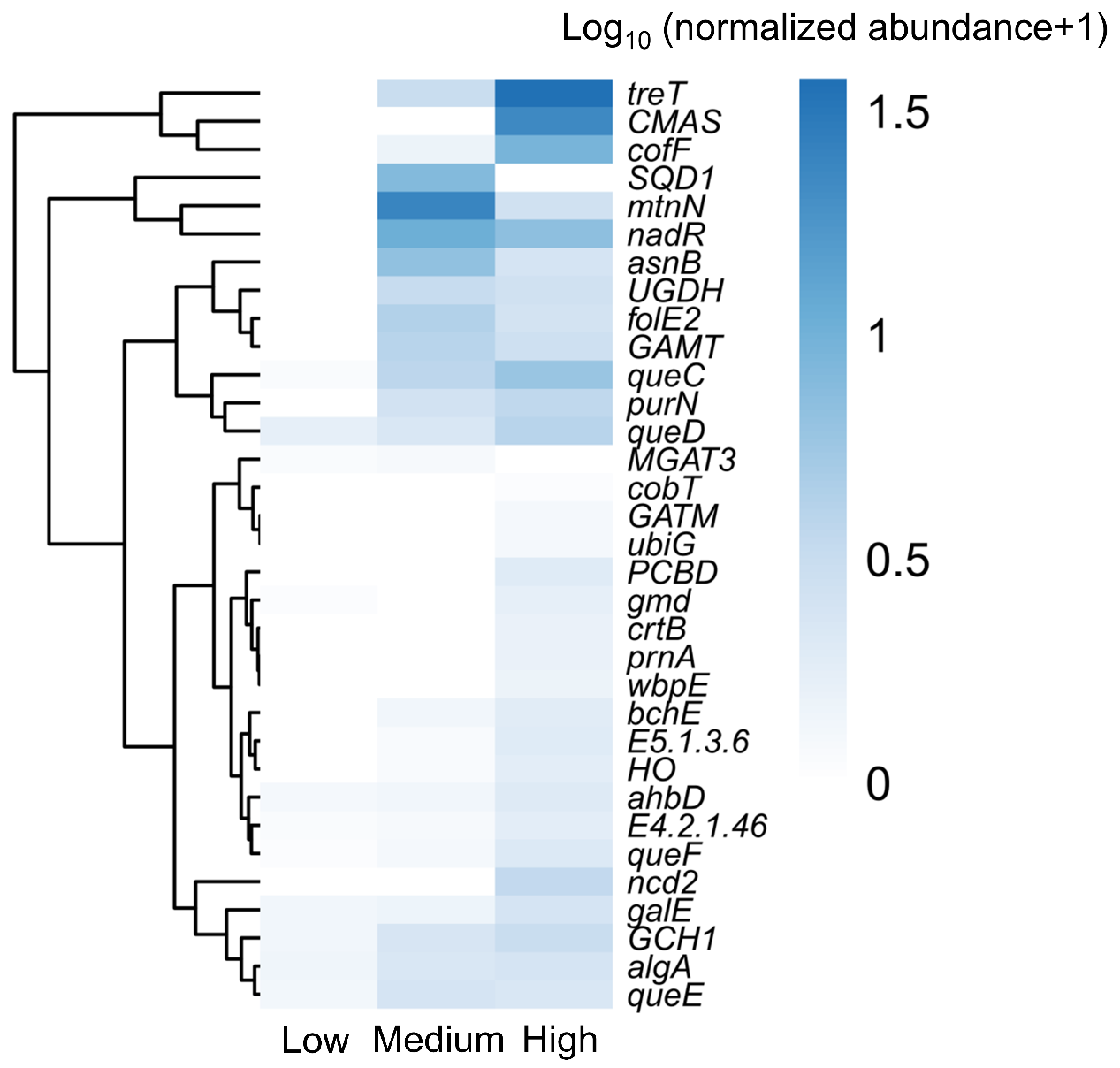


**Figure S9** Heatmap showing significantly differentially expressed viral AMGs across low-, medium-, and high-salinity regions based on metatranscriptomic data.

**Figure S10**


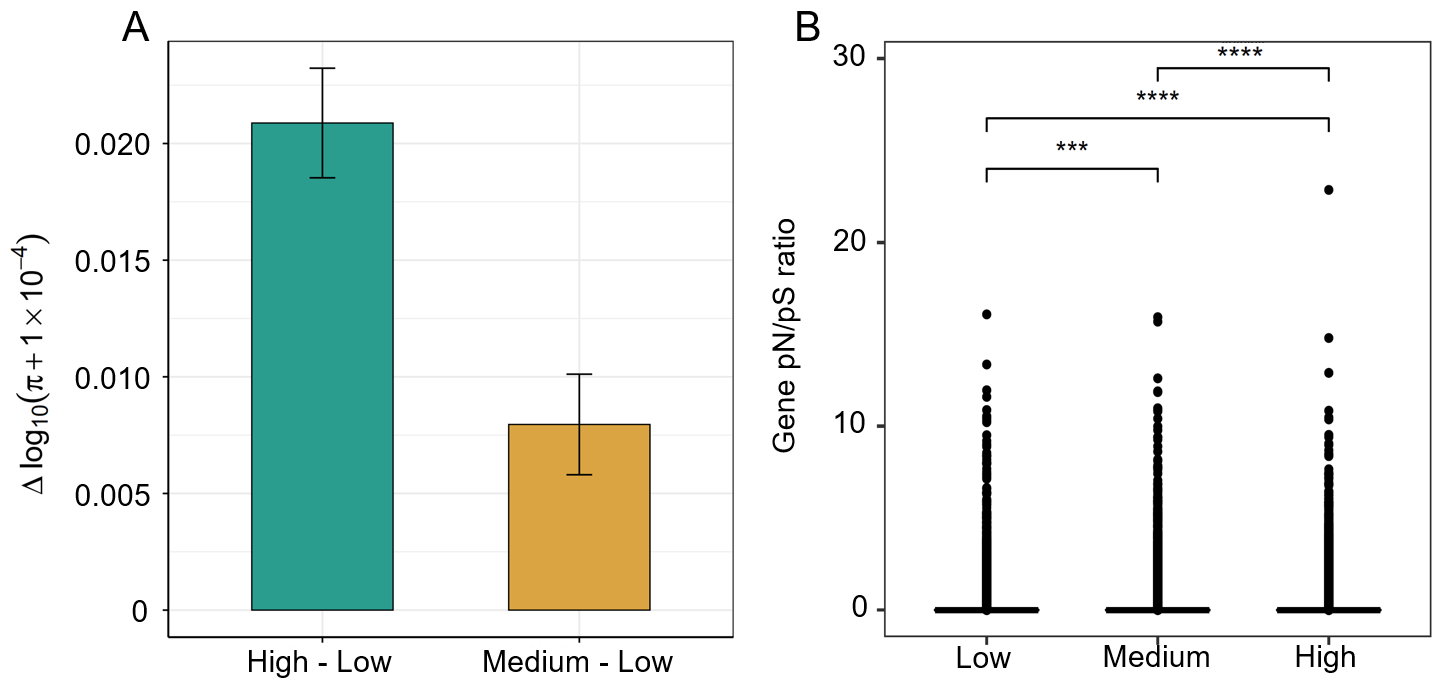


**Figure S10** Gene-level selection pressure indicated by pN/pS ratios of viral genes across salinity zones.

**Figure S11**


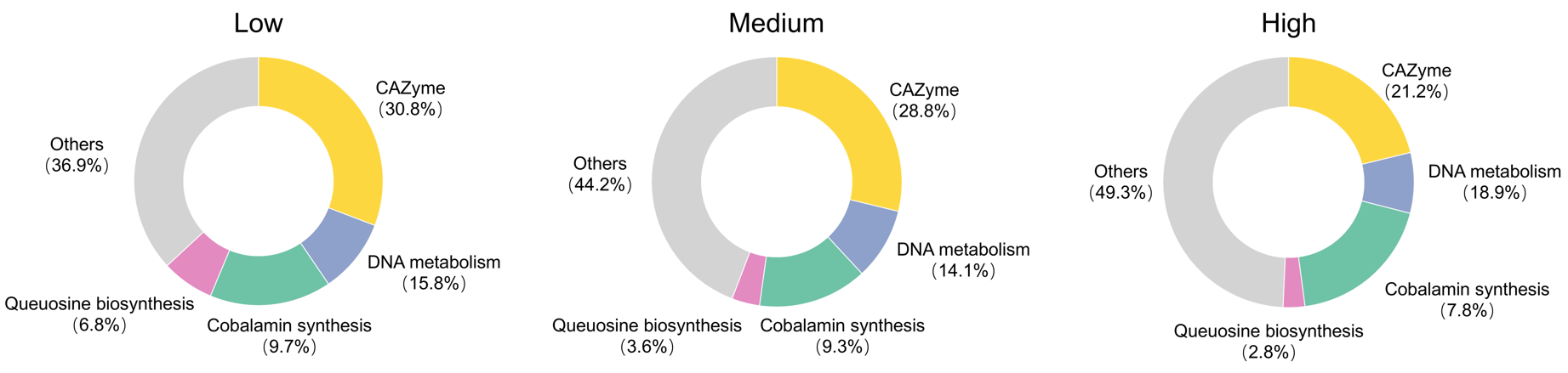


**Figure S11.** Functional classification of viral genes under positive selection (i.e., pN/pS ≥ 1) across salinity gradients.
